# Rapid hydrogel-based phage susceptibility test for pathogenic bacteria

**DOI:** 10.3389/fcimb.2022.1032052

**Published:** 2022-12-07

**Authors:** Sheetal Patpatia, Eric Schaedig, Anna Dirks, Lauri Paasonen, Mikael Skurnik, Saija Kiljunen

**Affiliations:** ^1^ Human Microbiome Research Program, Faculty of Medicine, University of Helsinki, Helsinki, Finland; ^2^ UPM Biomedicals, Helsinki, Finland; ^3^ Division of Clinical Microbiology, HUSLAB, Helsinki University Hospital, Helsinki, Finland

**Keywords:** bacteriophage, phagogram, phage therapy, hydrogel, rapid test, diagnostics, phage susceptibility, personalized medicine

## Abstract

Phage therapy is one alternative to cure infections caused by antibiotic resistant bacteria. Due to the narrow host range of phages, hundreds to thousands of phages are required to cover the diversity of bacterial pathogens. In personalized phage therapy, fast selection of the phages for individual patients is essential for successful therapy. The aims of this study were to set up a rapid hydrogel-based liquid phage susceptibility assay (PST) for the selection of phages for therapeutic use and to establish a “ready-to-screen” plate concept, where phages are readily stored in hydrogel as small droplets in microtiter plate wells. We first tested four commercially available hydrogels (GrowDex, Askina, Purilon, and Intrasite) for their suitability as phage matrices in PSTs with four phages, two of which infecting *Escherichia coli* and two *Staphylococcus aureus*. Of these four hydrogels, GrowDex was the best matrix for PST, as it did not inhibit bacterial growth, released phages quickly when mixed with bacterial culture, and maintained phage viability well. We then optimized the assay for both optical density and microscopy readers using GrowDex as matrix with 23 bacterial strains representing 10 different species and 23 phages possessing different morphologies and genome sizes. When the bacterial growth was monitored by microscopy reader, the PST was executed in just 3 hours, and there was no need for overnight culturing bacterial cells prior to the assay, whereas using optical density reader, bacteria had to be pre-cultured overnight, and the assay time was five hours. Finally, we evaluated the effect of three different chemical stabilizers (trehalose, hyaluronic acid, and gelatin) in a six-month stability assay with six model phages. These phages assay behaved very differently in respect to the chemical stabilizers, and there was not a single stabilizer suitable for all phages. However, when gelatin (0.01%) or hyaluronic acid (0.2 mg/ml) was used as stabilizer, all tested phages were still considered as positives in PST after a six-month storage in 1 ml volume. In “ready-to-screen” plates, the differences in phage stabilities were even more profound, varying from two to six months for the most and least stable phages, respectively.

## 1 Introduction

Antibiotic-resistant pathogenic bacteria, such as ESKAPEE-pathogens (*Enterococcus faecium, Staphylococcus aureus, Klebsiella pneumoniae, Acinetobacter baumannii, Pseudomonas aeruginosa, Enterobacter* spp., and *Escherichia coli*), cause major challenges for clinical settings (Pendleton et al., 2013; Mulani et al., 2019). The World Health Organization (WHO) has declared antibiotic resistance as one of the major global health threats of our time (WHO, 2019). Because of the emergence of antibiotic resistance, there is an urgent need to find alternative antimicrobial medications and treatments (Duval et al., 2019; WHO, 2021; Murray et al., 2022). One such emerging antimicrobial treatment is phage therapy, the use of bacteriophages (phages), viruses that infect bacteria, to clear bacterial infections (Clokie MR et al., 2011). Phages are the most ubiquitous biological entities on Earth and can be found in diverse environments (Keen, 2015). The host specificity of phages is very narrow; therefore, phages used for therapy can be selected in such a way that they target pathogenic bacteria without impacting the microbiota of the human host. Various studies in the past ten years have proven phages safe for treating humans (Abedon et al., 2011; Pires et al., 2015; Chan et al., 2018; Fish et al., 2018; Jault et al., 2019; Zalewska-Piatek and Piatek, 2020). Phages were discovered more than a hundred years ago, but became less commonly used in treating bacterial infections due to the broad success of antibiotics (Chanishvili, 2012). However, with antibiotic resistance now on the rise, interest in phage therapy is once again increasing and various methodologies are being developed for the efficient clinical use of phages (Ács et al., 2020; Reuter and Kruger, 2020).

A major obstacle in modernizing phage therapy is the high level of specificity of phages for their bacterial hosts, which necessitates the use of a large number of phages to cover the broad spectrum of pathogenic bacteria (Pirnay et al., 2019). Clinical professionals may circumvent this issue by using personalized phage therapy, in which a therapeutic phage cocktail is prepared individually for each patient based on the phage susceptibility of the infecting bacterium. The first steps in personalized phage therapy are the isolation of the pathogenic bacterial strain and the selection of suitable phages to be used therapeutically (Reuter and Kruger, 2020). The latter requires screening of the isolated bacterium with a phage susceptibility test (PST) to identify infective phages from a large phage collection as quickly as possible. Rapid diagnostic testing for phage susceptibility is thus an emerging branch of interest among the phage research community and clinicians, and the establishment of standardized methods for the identification of suitable phages for personalized phage therapy is a major challenge (Suh et al., 2022).

Conventional PST methodologies have not seen major changes since the discovery of phages. Traditional methods (*i.e*., the double-layer agar assay) typically require between 24 to 48 hours and may take even longer depending on the size of the phage collection and the number of clinical bacterial isolates to be screened (Rajnovic et al., 2019). Some automated tools for plaque analysis and quantification have been published (Trofimova and Jaschke, 2021), but otherwise, the options for automation, and high throughput are still quite limited. These methods thus still largely depend on manual work, which makes them slow and labor intensive (Henry et al., 2012). These screening processes thus slow the preparation of therapeutic phage cocktails for patients who are often in critical condition. PST methods based on liquid culture may offer better possibilities for process automation and high throughput screening. However, even these methods are often laborious and require the maintenance of viable high-titer phage stocks that can be rapidly used for the screening.

The challenges in testing a large number of phages quickly are immense, and developing standardized, fast high throughput phage screening methods is essential (Daubie et al., 2022). There have been few studies on developing fast and automatable PSTs, some of which use complex ways to examine phage susceptibility. For example, Low et al. described a method that identifies phage- host interactions by fluorescence tagging of phages with subsequent tracking of the infection using flow cytometry and confocal fluorescence microscopy, along with live/dead assay for phage susceptibility (Low et al., 2020). There are also studies where the prediction of phage infectivity was approached through machine learning and imaging (Perlemoine et al., 2021; Lood et al., 2022; O'Connell et al., 2022). The study by Perlemoine et al. introduced a custom wide-field lens-less imaging device, which can be used for phage plaque detection (Perlemoine et al., 2021). The methodology can detect phage-resistant bacterial micro-colonies within 3 hours, however, the scalability to screen multiple phages has not yet been demonstrated. More recently, a study utilizing surface plasmon resonance imaging (SPRi) and phase imaging for phage susceptibility testing demonstrated phage detection in less than two hours (O'Connell et al., 2022). However, the proof-of-concept study was performed only with *Staphylococcus aureus* and *Pseudomonas putida* bacterial species. The challenge with many of these novel methods is that they require expensive, and sometimes custom designed devices, which makes them inaccessible to many phage laboratories. Furthermore, they still require large-scale testing with a vast number of phages infecting several different bacterial species before their universalization.

There are also PSTs that are simple and scalable for both small and large laboratories. These assays are often based on the measurement of optical density (Cooper et al., 2011; Xie et al., 2018; Rajnovic et al., 2019). However, even though various bacterial concentrations with multiple phage titers were tested in these studies, they only focused on few bacterial species. Thus, these methods still require standardization and universalization through systematic validation.

To optimize the efficiency of personalized phage therapy, the diagnostics step of the pipeline should be scalable, robust, and accessible for laboratories of all sizes. In this study, we aimed to create a rapid and robust PST, which can be used universally for many clinically relevant bacterial species. To this end, we developed a hydrogel-based liquid PST and optimized it with 23 bacterial strains representing 10 different species and 23 phages possessing different morphologies. The assay was further validated with 20 additional *Pseudomonas aeruginosa* strains and four *P. aeruginosa* -specific phages. We used two different approaches, optical density and microscopy, to measure bacterial growth upon phage infection. This hydrogel-based assay offers a fast and automation-friendly method for both large- and small-scale commercial, research, and clinical laboratories, enhancing the selection of the appropriate phages for personalized cocktail preparation and reducing the bench-to-bedside time of phage therapy.

## 2 Materials and methods

### 2.1 Bacterial strains, phages, and propagation

Bacterial strains used in the study are described in **Table 1**, and phages used in the study are described in **Table 2**.

**Table 1:** Bacterial strains used in the work.

Species	Strain code	Source/Reference
*Acinetobacter baumannii*	#5542	HUSLAB
	#5707	HUSLAB
*Acinetobacter pittii*	#5565	(Pulkkinen et al., 2019)
*Enterobacter cloacae*	#5508	HUSLAB
	#5532	HUSLAB
*Enterococcus faecalis*	#5498	EPSHP
	#6569	HUSLAB
*Enterococcus faecium*	#5543	HUSLAB
*Escherichia coli*	#5507	HUSLAB
	#5521	HUSLAB
*Klebsiella pneumoniae*	#6037 (ATCC 43816)	ATCC
	#6815	(Townsend et al., 2021)
	#6740	(Yerushalmy et al., 2019)
*Pseudomonas aeruginosa*	#5525	HUSLAB
	#6327 (PA1)	(Mattila et al., 2015)
	#6329 (PA8)	(Mattila et al., 2015)
	#6331 (PA11)	(Mattila et al., 2015)
	#6663	HUSLAB
	#6704 (IATS O1)	(Lam et al., 2011)
	#6705 (IATS O2)	(Lam et al., 2011)
	#6706 (IATS O3)	(Lam et al., 2011)
	#6707 (IATS O4)	(Lam et al., 2011)
	#6708 (IATS O5)	(Lam et al., 2011)
	#6709 (IATS O6)	(Lam et al., 2011)
	#6710 (IATS O7)	(Lam et al., 2011)
	#6711 (IATS O8)	(Lam et al., 2011)
	#6712 (IATS O9)	(Lam et al., 2011)
	#6713 (IATS 10)	(Lam et al., 2011)
	#6714 (IATS 11)	(Lam et al., 2011)
	#6715 (IATS 12)	(Lam et al., 2011)
	#6716 (IATS 13)	(Lam et al., 2011)
	#6717 (IATS 14)	(Lam et al., 2011)
	#6718 (IATS 15)	(Lam et al., 2011)
	#6719 (IATS 16)	(Lam et al., 2011)
	#6720 (IATS 17)	(Lam et al., 2011)
	#6721 (IATS 18)	(Lam et al., 2011)
	#6722 (IATS 19)	(Lam et al., 2011)
	#6723 (IATS 20)	(Lam et al., 2011)
	#6728	HUSLAB
	#6886	MVZ
*Staphylococcus aureus*	#5676 (13KP)	(Leskinen et al., 2017)
	#6433 (19A2)	(Tuomala et al., 2021)
*Staphylococcus xylosus*	#6743	(Oduor et al., 2019)

HUSLAB; Division of Clinical Microbiology, the Hospital District of Helsinki and Uusimaa, Finland, EPSHP; The Hospital District of South Ostrobothnia, Finland, MVZ; MVZ Laborarztpraxis Dres. med. Jochem, Walther und Kollegen MVZ GbR, Germany.

**Table 2:** Bacteriophages used in the work.

Phage	Host strain	Source/Reference	Morphotype, Genome size
vB_AbaA_fBenAci001 (fBenAci001)	*A. baumannii* #5542	GenBank: MW056501.1	P; 40.5 kb
vB_AbaA_fBenAci002 (fBenAci002)	*A. baumannii* #5707	GenBank: MW056502.1	P; 42.0 kb
vB_ApiM_fHyAci03 (fHyAci03)	*A. pittii* #5565	(Pulkkinen et al., 2019)	M; 166.0 kb
fHe-Ecl05	*E. cloacae* #5508	Own collection^2^	P; 38.3 kb
fOu-Ecl03	*E. cloacae* #5532	Own collection^2^	M; 84.3 kb
F-Mali04	*E. faecalis* #5498	Own collection^2^	S. 41.3 kb
fGPio-Efa01	*E. faecalis* #6569	Microgen, Russia^1^	ND
fHo-Efm06	*E. faecium* #5543	Own collection^2^	M; 156.6. kb
vB_EcoA_fTuEco01 (fTuEco01)	*E. coli* #5507	GenBank: MZ031013.1	P; 44.3 kb
vB_EcoM_fHoEco02 (fHoEco02)	*E. coli* #5521	(Kiljunen et al., 2018)	M; 167.0 kb
vB_EcoS_fPoEco01 (fPoEco01)	*E. coli* #5521	GenBank: MT711526.1	S; 44.4 kb
KpGranit	*K. pneumoniae* #6740	(Yerushalmy et al., 2019)	S; 122.7 kb
vB_KoM-Pickle (Pickle)	*K. pneumoniae* #6815	(Townsend et al., 2021)	M; 175.2 kb
fRu-Kpn01	*K. pneumoniae* #6037	Microgen, Russia^1^	P; 40.9 kb
PaP1_EPu-2019 (PA1P1)	*P. aeruginosa* #6327	(Mattila et al., 2015), GenBank: MN131141.1	M; 66.6 kb
PA8P1	*P. aeruginosa* #6329	(Mattila et al., 2015), GenBank: MN131142.1	M; 65.7 kb
PA11P1	*P. aeruginosa* #6331	(Mattila et al., 2015), GenBank: MN131143.1	M; 66.0 kb
vB_PaeM_fHoPae01(fHo-Pae01)	*P. aeruginosa* #6663	GenBank: MK318076.1	M; 66.4 kb
fGPio-Pae001A	*P. aeruginosa* #6886	Microgen, Russia^1^	P; 45.3 kb
fMGyn-Pae01	*P. aeruginosa* #6728	Micromir, Russia^1^	M; 277.9 kb
vB_SauM_fRuSau02 (fRuSau02)	*S. aureus* #5676	(Leskinen et al., 2017)	M; 148.5 kb
vB_SauP_EBHT (mEBHT)	*S. aureus* #6433	GenBank: MT926124.1	P; 17.5 kb
Stab21	*S. xylosus* #6743	(Oduor et al., 2019)	M; 154.0 kb

P; Podovirus morphotype, M; Myovirus morphotype, S; Siphovirus morphotype, ^1^Isolated from a commercial phage cocktail using indicated host strain, ^2^Isolated from wastewater using indicated host strain. ND, Not determined.

Bacterial strains were propagated in liquid culture using Lysogeny Broth (LB), (Sambrook, 2001) at 37°C. When needed, LB was solidified with 1.5% or 0.4% (w/v) agar, to prepare Lysogeny agar (LA) or soft-agar, respectively.

For phage propagation, a single bacterial colony streaked from a LA plate was inoculated into 5 ml fresh LB and incubated at 37°C and 200 rpm, overnight. The phage lysate was prepared in 10 ml fresh LB broth by adding 400 μl overnight bacterial culture and 40 μl of phage stock aliquoted from 20% glycerol stocks stored at -70°C. The culture was then incubated at 37°C, 200 rpm, and once the culture had cleared (~5 hours), the lysate was centrifuged at 3,000 x g, 4°C for 20 min, after which the supernatant was filtered using 0.22 μm Minisart RC Syringe Filter (Sartorius, Germany). Finally, sucrose was added to a final concentration of 8% (w/v) and the lysates were stored at 4°C until further use.

### 2.2 Phage titration by double-layer agar method

To determine the titer of the phage samples, the double-layer agar plating method described elsewhere (Sambrook, 2001) with slight modifications was used. Briefly, bacterial logarithmic culture was prepared by growing the cells in 1.3 ml LB for 2 to 3 h. Optical density was measured with DSM Cell Density Meter (Laxco, USA), and the volume of bacterial culture used for titration plate was calculated from equation µl = 45 ÷ A600. Bacteria and phage dilutions were added into 3 ml soft agar temperated to 55°C and poured onto LA plates. The plates were left to solidify at room temperature (RT) for 30 minutes and incubated at 37°C overnight. The formed plaques were counted to determine the titer of the phage, expressed as plaque forming units (PFU) per ml. The phage titrations were done in triplicate, and mean values and standard deviations (SD) were calculated. Efficiency of plating (EOP) was calculated by determining EOP value 1 as the phage titer in its original isolation host and setting its titers in other hosts in proportion to it.

### 2.3 Determination of bacterial colony forming units

Colony forming units (CFU) of the bacterial cultures were determined for all the bacterial species used in the study. 100 µl of serially diluted culture was plated on LA plate and incubated at 37°C overnight. Formed colonies were counted and CFU/ml was determined. Measurements were performed in triplicates and mean values were calculated.

### 2.4 Phage susceptibility assay by liquid culture

Phage infection was measured by following bacterial growth in liquid culture. The assay was optimized and validated for 43 bacterial strains (**Table 1**) and their respective phages (**Table 2**) for both optical density and microscopy readers.

#### 2.4.1 Assay with optical density reader

For the measurement on absorbance plate readers, Hidex Sense microplate reader (Hidex, Finland), and Bioscreen C analyzer (Growth Curves AB Ltd, Finland) were used. Phage lysates (10^8-9^ PFU/ml) in 10 µl droplets were added into the bottom of the 96-well microtiter-plate (SpectraPlate-96 TC, PerkinElmer, USA) or Honeycomb2-plate (Growth Curves AB Ltd) for Hidex and Bioscreen C analyzer, respectively. 200 µl of diluted overnight bacterial culture was then added. As control, 200 µl of bacterial dilution was added into the wells along with 10 µl LB. The plates were incubated at 37°C and 200 rpm, and bacterial growth was monitored by measuring absorbance at 600 nm of each well at one-hour intervals, until the stationary phase of the bacteria was reached. For each bacterial species, dilutions of 1:10 to 1:500 were first tested without phage to find the optimal starting dilution for the assay. A phage was considered infectious toward a given bacterial strain if the culture absorbance was < 50% of the control without phage after 5 hours. The phage was considered partially infectious if bacterial growth was between 50% and 70% in respect to the control and noninfectious (negative) if bacterial growth was higher than 70% of the control. For all other bacterial species except *P. aeruginosa*, samples were analyzed in triplicate and mean and SD were calculated to ensure the validity of the results. For *P. aeruginosa*, samples were analyzed as duplicate, and mean was calculated.

#### 2.4.2 Assay with microscopy reader

The microscopy plate reader, oCelloScope (BioSense Solutions Aps, Denmark), was used to measure bacterial growth with a liquid growth assay. To test the effect of bacterial starting concentration and growth phase in the assay, bacterial overnight pre-culture in 5 ml LB, two-hour pre-culture prepared in 1.3 ml LB, and bacteria directly picked from the plate and inoculated into 1 ml LB were used. Bacterial culture absorbance (OD_600_) was adjusted to 0.35 as measured with a DSM Cell Density Meter and diluted 1:20 to 1:1000 with LB. Phage lysates (10^5-9^ PFU/ml) in 10 µl droplets were added into the bottom of 96-well microtiter-plates followed by 200 μl of the diluted bacterial culture. As control, 200 µl of bacterial culture was added into the wells along with 10 µl LB. Plates were covered with qPCR adhesive clear plate seals (Azenta Life Sciences, UK) and incubated in oCelloScope at 37°C without shaking. Images were acquired every 30 minutes up to ten hours. Each sample was analyzed in triplicate and mean and SD were calculated. Results were interpreted as earlier.

### 2.5 The use of hydrogels as phage matrix in phage susceptibility assay

Four commercially available hydrogels Askina (B.Braun, Germany), Purilon (Coloplast, Denmark), Intrasite (Smith&Nephew, UK), and GrowDex (UPM Biomedicals, Finland) were tested as matrices for PST assay. Detailed information of the conditions used is shown in **Table 3**.

**Table 3:** Hydrogels and chemical stabilizers used in the work.

Hydrogel/Reagent	Composition	Concentrations used	Producer
*GrowDex*	Wood-derived nanofibrillar cellulose and ultra-pure water		UPM Biomedicals
*Askina*	Purified water, glycerol, disodium EDTA, Carbopol 940, sorbital		B.Braun
*Purilon*	Purified water, sodium carboxymethylcellulose, and calcium alginate		Coloplast
*Intrasite*	Carboxymethylcellulose polymer 2.3%, propylene glycol 20%, water		TJ Smith&Nephew
*Trehalose*		3.3%, 2.0%, 1.0%	Fisher BioReagents
*Hyaluronic acid*		0.2 mg/ml, 1.0 mg/ml	Sigma
*SMG-Buffer*	100 mM NaCl, 10mM MgSO4, 50 mM Tris-HCl, pH 7.5, 0.01% (w/v) gelatin	0.01% of gelatin in SM-Buffer	(Sambrook, 2001)
*SM-Buffer*	100 mM NaCl, 10mM MgSO4, 50 mM Tris-HCl, pH 7.5		(Sambrook, 2001)

Phage-hydrogel mixtures were prepared in 1 ml volume by mixing 333 µl of phage dilution (3x10^8-9^ PFU/ml) prepared in SMG-Buffer (**Table 3**) and 667 µl of (pre-diluted) hydrogel. The final concentration of the phages in the mixture was 1x10^8-9^ PFU/ml. The viability of phages in the mixtures was verified by titration by double-layer agar method and the suitability of the phage-hydrogel mixture in the phage susceptibility assay was evaluated by optimized liquid culture assays with both optical density and microscope readers as described above.

### 2.6 Phage stability in GrowDex

Phage stability experiments were conducted with phage-GrowDex mixtures with and without the addition of chemical stabilizers. Three chemical stabilizers including trehalose, hyaluronic acid, and gelatin were used, and concentrations are detailed in **Table 3**. The mixtures were prepared using SM-buffer (**Table 3**) as diluent. The compositions were prepared in 1 ml volume by first mixing the stabilizer and SM buffer, and then adding 327 µl of this composition into 663 µl of GrowDex hydrogel (1.5% stock concentration). 10 µl of phage dilution (3x10^8-11^ PFU/ml) was then added to hydrogel-stabilizer mixture. The final concentration of phages in the mixtures was 1x10^8-11^ PFU/ml and the final concentration of GrowDex was 1.0%.

#### 2.6.1 Phage stability in tubes

The phage-hydrogel compositions were stored at 4°C in 2 ml screw cap tubes for six months. The stability of phages in the mixtures was monitored by titrating the compositions after 2 hours, 1 week, and 1, 2, 4, and 6 months after the preparation of the mixtures.

#### 2.6.2 Phage stability on microtiter well plates

“Ready-to-screen plates” were prepared by adding 10 µl of phage-hydrogel compositions on the bottom of the wells of either microtiter or honeycomb-plates. The plates were then sealed with PCR Foil Seal (Azenta Life Sciences) and covered with lids. The ready-to-screen plates were stored at 4°C for six months, and the phage susceptibility assay was conducted immediately and 1 week, 1, 2, 4, and 6 months after the preparation of the plates using the optimized liquid culture method as described above.

#### 2.6.3 Transportation of ready-to-screen plates

Two sets of identical ready-to-screen plates were prepared as described earlier. One set of copies was measured by phage susceptibility assay using absorbance reader immediately after the preparation, and one set was sent *via* local post or courier service to ourselves and to Leibniz-Institut Deutsche Sammlung von Mikroorganismen und Zellkulturen GmbH (DSMZ), Germany, respectively. The transported plates were used for PST approx. 1 week after the preparation of the plates.

### 2.7 Graphical representations and statistical analysis

Graphical representations and calculations of mean and standard deviation (SD) values were conducted with OriginPro 2021b program (OriginLab). Statistical analyses were performed with one-way ANOVA in OriginPro and probability values (*p*) lower or equal to 0.01 were considered significant.

## 3 Results

### 3.1 Optimizing liquid growth assay for rapid phage susceptibility testing

Liquid growth assay is a widely used method for PST. However, even small changes in bacterial growth conditions may affect the results; therefore, the assay needs to be optimized and validated to prevent person-to-person variation in the interpretation of the results. To this end, we first optimized the assay for growth conditions for 23 bacterial strains representing *Staphylococcus* spp.*, Acinetobacter* spp.*, Escherichia coli, Klebsiella pneumoniae, Pseudomonas aeruginosa, Enterococcus* spp., and *Enterobacter cloacae* (**Table 1**, **Table 2**). Our aim was to find conditions, wherein bacteria would exhibit optimal logarithmic growth without yet entering stationary phase during the PST, to ensure efficient phage infection. The purpose was to be able to measure the phage susceptibility within one working day. As we aimed for the assay to measure phage infection and not lysis from without, we reasoned that the assay time needs to allow at least two lytic cycles of phages. Therefore, we set the minimum time allowed for the bacteria to reach stationary phase to two hours. We set up the assay conditions for two different types of growth measurement techniques, optical density and microscopy readers.

**Figure 1:**
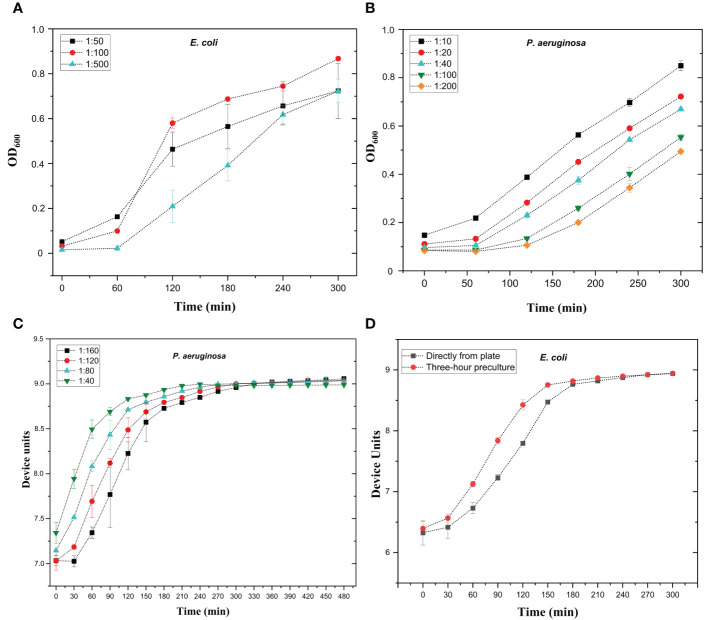
Optimization of bacterial growth curves with optical density reader **(A, B)** and microscopy reader **(C, D)**. **(A)** Growth curves of *E*. *coli* strain #5506 in absorbance reader. Overnight bacterial culture was diluted in lysogeny broth (LB) to 1:50, 1:100, and 1:500 dilutions. Bacterial dilutions were added in 200 µl volumes into microtiter well plate and bacterial growth was measured using absorbance reader with optical density (OD) at 600 nm at one-hour intervals for 5 hours. **(B)** Growth curves of *P. aeruginosa* strain #6329 in absorbance reader. Overnight bacterial culture was diluted in lysogeny broth (LB) to 1.10, 1:20, 1:40, 1:100, and 1:200 dilutions. Bacterial dilutions were added in 200 µl volumes into microtiter well plate and bacterial growth was measured as in A. **(C)** Growth curves of *P. aeruginosa* strain #6329 with oCelloScope microscope. Overnight bacterial culture was diluted in LB to 1:40, 1:80, 1:120, and 1:160. Diluted culture was added in 200 µl volume into the microtiter well plate. Bacterial growth was followed at 30-minute intervals for eight hours. **(D)** Growth curves of *E*. *coli* strain #5521 with microscopy reader. Bacteria were either pre-cultured for three hours or picked directly from plate and suspended in LB. The OD of the 3-hour pre-culture was adjusted to 0.35 and further diluted 1:200, and 200 µl of the dilution was aliquoted into microtiter plate wells. Bacterial growth was followed at 30-minute intervals for five hours. Device units indicate the microscopy reader values. Each sample was measured in triplicate; mean values and SD are indicated.

To optimize the bacterial concentration in the assay, different dilutions of overnight cultured bacteria were used to create growth curves, and the aim was to achieve logarithmic growth from two to five hours. The optimal dilutions varied from species to species with both OD and microscopy reader. With the OD reader, the overnight culture dilution range was from 1:500 for *E. coli* and *K. pneumoniae* to 1:40 for *P. aeruginosa* (**Table 4**). Representative images of growth curves of *E. coli* and *P. aeruginosa* are shown in **Figure 1A, B**, respectively. The typical bacterial concentrations in overnight cultures of seven representative strains were 10^9-10^ CFU/ml, indicating that the starting cell counts in the OD reader assays ranged from 1.5 × 10^6^ CFU to 3.0 × 10^7^ CFU for *E. coli* and *E. faecalis*, respectively (**Table S1**). With microscopy reader, the optimal dilutions were between 1:100 to 1:200, when starting from o/n culture standardized to OD 0.35 as measured with a DSM Cell Density Meter (**Table 4**, **Figure 1C**). The bacterial concentrations in OD 0.35 cultures were approx. 10^8^ CFU/ml (**Table S1**). This indicates that starting cell counts for microscopy reader assay were 1 × 10^5^ to 1 × 10^6^ CFU, depending on the species (**Table S1**).

**Table 4:** Species-specific optimizations for phage susceptibility testing*.

Species	Dilution from O/N culture	Phage (PFU/well)	Screening time	Dilution from OD_600_ 0.35	Phage (PFU/well)	Screening time
	Absorbance reader			Microscopy reader		
*Staphylococcus* spp.	1:100	10^6^	5 hours	1:200	10^5^	3 hours
*Acinetobacter* spp.	1:100	10^6^	5 hours	1:200	10^5^	4 hours
*Escherichia coli*	1:500	10^6^	5 hours	1:200	10^3^	3 hours
*Klebsiella Pneumoniae*	1:500	10^6^	5 hours	1:200	10^5^	3 hours
*Pseudomonas aeruginosa*	1:40	10^7^	5 hours	1:100	10^5^	4 hours
*Enterococcus* spp.	1:50	10^7^	5 hours	1:100	10^4^	4 hours
*Enterobacter cloacae*	1:100	10^6^	5 hours	1:200	10^5^	3 hours

*The conditions were validated with all phages indicated in **Table 2**.

The enhanced sensitivity of microscopy reader allows it to record much smaller changes in bacterial concentration than conventional absorbance readers. Therefore, we aimed to test whether the microscopy reader could track logarithmic growth curves (and therefore detect phage infection) without the use of overnight bacterial cultures. To this end, bacteria diluted from overnight culture, three-hour pre-culture, and directly picked from plate cultures were used to inoculate the growth curve. The starting OD prior to dilution was standardized to 0.35. We noticed no difference between growth curves inoculated from overnight and three-hour cultures (not shown), but there was a slight delay in growth when bacteria were picked directly from plates (**Figure 1D**). However, even with the delay, all tested species except *E. faecalis* performed logarithmic growth curves within 3 to 4 hours and can be used without any pre-culture step. For *E. faecalis*, a pre-culture of 2 to 3 hours is recommended. Optimized conditions for bacterial growth curves are shown in **Table 4**.

We next wanted to optimize phage concentration in the assay. To this end, we tested phage infection with phage titers between 10^3^ to 10^7^ PFU/well. The optimal phage titer differed slightly between different host species, with *P. aeruginosa* and *E. cloacae* requiring ten-fold higher phage concentrations than other species (**Table 4**). An example of phage optimizing curves is shown in **Figure S1**. The PST measured with OD readers required 10^6^ or 10^7^ PFU/well, whereas the assay measured with the microscopy reader was successful with ~100-fold fewer phages. The input multiplicity of infection (MOI) values calculated for representative strains for both OD reader and microscopy reader ranged from 2×10^-3^ to 9×10^-1^ (**Table S1**).

**Figure 2:**
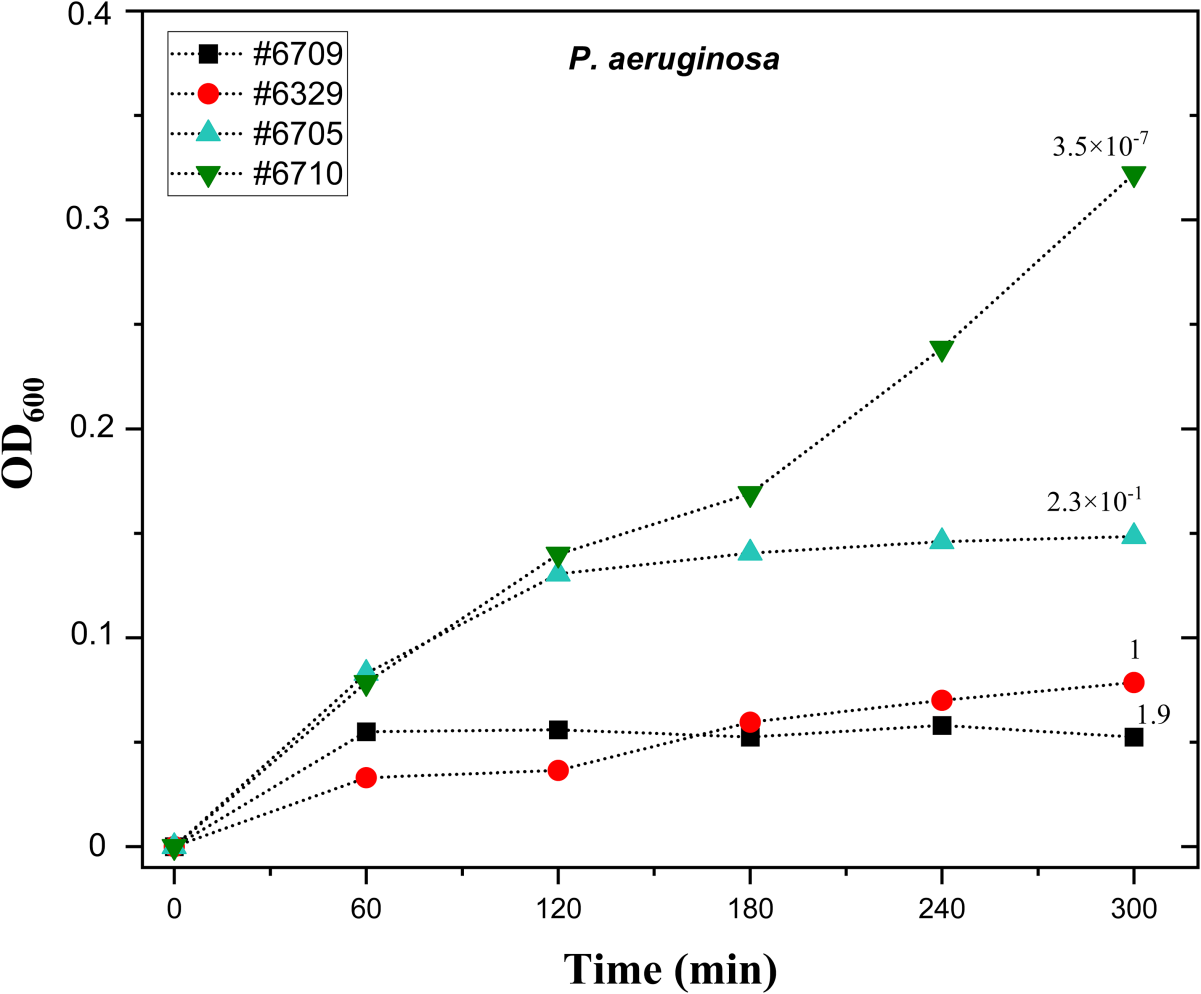
The correlation of liquid phage susceptibility test and efficiency of plating with phage PA8P1. Ten µl droplets of PA8P1 (10^9^ PFU/ml) were transferred onto the bottoms of microtiter plate wells. Overnight cultures of *P. aeruginosa* strains #6709, #6329, #6705, and #6710 were diluted 1:40, and 200 µl of the bacterial dilutions were added into the wells. The plate was incubated at 37°C and 200 rpm, and absorbance at 600 nm was measured at one-hour intervals. The numbers above each growth curve indicate efficiency of plating (EOP) of PA8P1 in the corresponding strain, and its EOP in the original isolation host #6329 is denoted as 1. Each sample was analyzed in duplicate, and the mean is shown.

To validate the selected phage titration for liquid-based PST, we tested the liquid assay with the PA8P1 phage (**Table 2**) and four *P. aeruginosa* strains for which PA8P1 has different infectivity, expressed as efficiency of plating (EOP). PA8P1 efficiently inhibited the growth of the original isolation host #6329 and strain #6709, with EOPs of 1 and 1.9, respectively (**Figure 2**). Strain #6705, in which the EOP was 2.3 × 10^-1^, was inhibited by ~60% when compared to strain #6710, for which the phage had an EOP of 3.5 × 10^-7^ and which served as phage-resistant control in this experiment. Strain #6705 would thus be considered as intermediately sensitive in our assay. Consequently, the phage infection curve correlated well with its EOP in the corresponding strain.

### 3.2 Hydrogel as phage protective matrix

**Figure 3:**
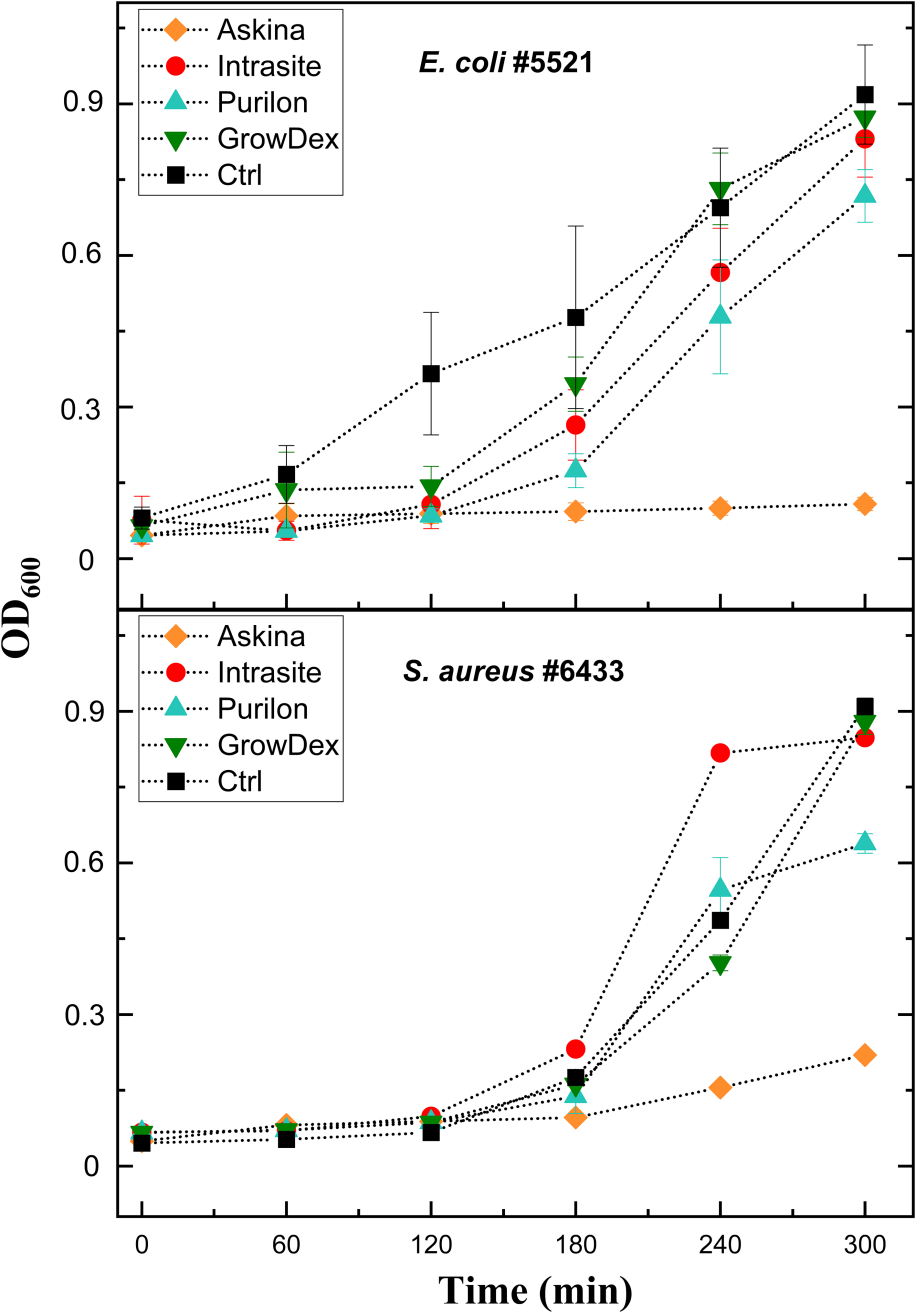
The effect of four commercial hydrogels on bacterial growth. Hydrogels Askina, Intrasite, Purilon, and GrowDex were added into bottom of the microtiter well plates as 10 µl droplets. Overnight cultures of *E. coli* strain #5521 and *S. aureus* strain #6433 were diluted 1:500 and 1:100, respectively. 200 µl of these bacterial dilutions were added into the wells, and the plate was incubated at 37°C and 200 rpm for five hours. Absorbance at 600 nm was measured at one-hour intervals. Bacteria grown in lysogeny broth without hydrogel served as control. Each sample was analyzed in triplicate, and the mean and SD are shown.

To enable the preparation of ready-to-screen PST microtiter plates and further reduce the time needed by labs to identify suitable therapeutic phages, we aimed to determine whether phages could be pre-aliquoted and stored in microtiter plates. Our preliminary experiments showed that all phages could not tolerate drying on the plates (not shown). We then aimed to test whether hydrogels could be used as protective matrices for phages. It was first important to identify hydrogels without antimicrobial properties, as such phage matrices would result in false positive results in PSTs. To this end, we tested the effect of four commercially available hydrogels, including Askina, Purilon, Intrasite, and GrowDex, on bacterial growth. The bacterial strains used in the experiments represented two clinically significant species, *E. coli* (strains #5521 and #5507) and *S. aureus* (#5676 and #6433). As seen in **Figure 3**, the Askina hydrogel inhibited bacterial growth in all tested strains, which suggested that it was antimicrobial and not suitable for PSTs. The Purilon hydrogel decreased the bacterial growth as well, but the effect was not as drastic. Intrasite and GrowDex hydrogels did not inhibit the growth of the tested strains, and we selected these two hydrogels for further experiments with phages.

**Figure 4:**
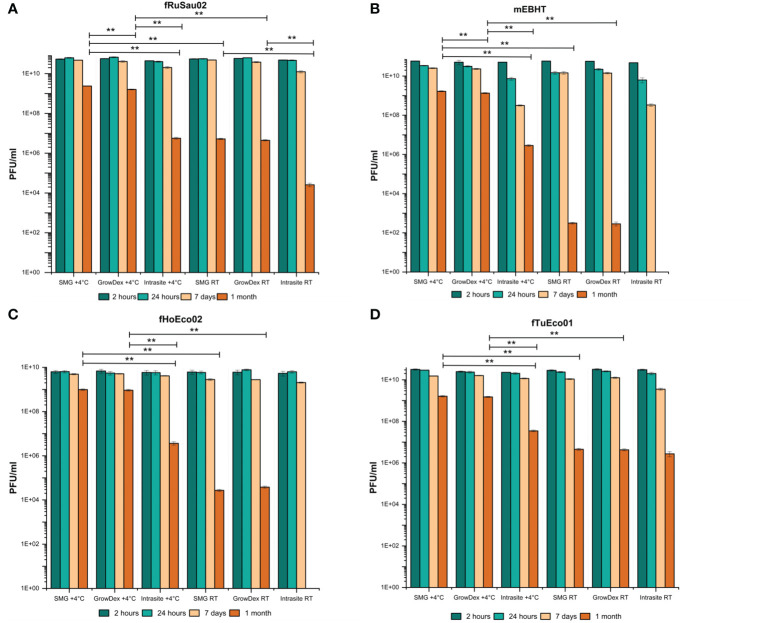
Comparison of Intrasite and GrowDex hydrogels as phage matrix. Phages fRuSau02 **(A)**, mEBHT **(B)**, fHoEco02 **(C)** and fTuEco01 **(D)** were stored with Intrasite and GrowDex-hydrogels at RT and 4°C in 2 ml tubes for up-to one month. Phage stability in hydrogels was assayed by titration with double-layer agar method after 2 and 24 hours, 7 days, and 1 month of storage. Bars and error bars indicate mean of three independent titrations and SD, respectively. The statistical analysis of one-month titers was made by one-way ANOVA test (**p ≤ 0.01). For phages mEBHT and fHoEco02, one-month result (no plaques) at RT in Intrasite was not included in statistical analysis.

To investigate whether Intrasite or GrowDex could be used as a phage matrix in PST, phages fHoEco02, fTuEco01, fRuSau02, and mEBHT (**Table 2**) were mixed with the hydrogels and stored for up to one month at RT and 4°C. These phages were selected as models, as they represent different genome sizes and two distinct morphological groups, and their hosts, *E. coli* and *S. aureus*, are pathogens representing gram-positive and gram-negative bacterial species. Phage titers were determined at 2-hour, 24-hour, 1-week and 1-month time points. In GrowDex and SMG buffer, all four phages showed similar stability pattern, whereas in Intrasite gel, phage stabilities were clearly lower than in the other two conditions tested (**Figure 4**). When stored at 4°C, phage titers in SMG and GrowDex were unchanged for one week, and dropped by approx. 10-fold in one month. In Intrasite, the titers of other phages except mEBHT remained stable for one week, but dropped by approx. 10^3^-fold, in one month. With the least stable phage, mEBHT, the titer decreased by 100-fold already in one week and by 10^4^-fold in one month (**Figure 4B**). Statistical analysis conducted with one-way ANOVA for titers at one-month time point of phages stored at 4°C showed significant difference between GrowDex and Intrasite for each phage. Statistical analysis also suggested that SMG was better storage medium than GrowDex for mEBHT and fRuSau02, but not for fTuEco01 and fHoEco02. However, even for mEBHT and fRuSau02, the actual differences in titers between SMG and GrowDex were marginal.

When stored at RT, phage stabilities were clearly lower than at 4°C. All tested phages were fairly stable for one week in SMG and GrowDex, whereas mEBHT showed ~1 log titer drop in Intrasite. However, in one month, phage titers suffered from a major titer decrease. In SMG and GrowDex, titers dropped by 10^4^-fold to 10^8^-fold, depending on the phage. There was no significant difference in the one-month stability between SMG and GrowDex for any of the phages. In Intrasite, phages fTuEco01 and fRuSau02 suffered from a 10^4^- and 10^6^-fold titer drop after one month, respectively. The one-month stability of fRuSau02 at RT was significantly lower in Intrasite when compared to either SMG or GrowDex, whereas fTuEco01 showed no difference between SMG, GrowDex, and Intrasite. Phages mEBHT and fHoEco02 totally lost their viability in Intrasite at RT. Consequently, we concluded that GrowDex hydrogel was better phage matrix than Intrasite hydrogel and that 4°C was superior storage temperature over RT. The remainder of the work was executed with GrowDex only.

As the preliminary experiment was performed with four phages only, we next aimed to verify the generality of GrowDex as a phage matrix. We tested the viability of twenty-three phages (**Table 2**) when mixed with GrowDex. We observed that all tested phages remained viable and inhibited the growth of their hosts after mixed with the hydrogel. Results of representative phages infecting each tested bacterial species are shown in **Figure S2**. Therefore, we concluded that GrowDex hydrogel can be used as general storage matrix for phages.

### 3.3 Phage long-term stability in GrowDex

#### 3.3.1 Long-term storage of the phage mixtures

After showing that GrowDex hydrogel can be used as a phage matrix in PST, we aimed to determine whether phages remain viable in it for longer than one month. To do so, we measured the viability of phages stored in GrowDex for a six-month period. We also wanted to test different chemical stabilizers in the storage matrices to determine whether they may enhance phage stability for long-term storage. The traditional stabilizer in phage buffers is 0.01% gelatin (Sambrook, 2001), which was also included in SMG buffer used as phage buffer in the short-term stability experiments in this work. The other stabilizers tested here included trehalose (3.3%, 2.0%, and 1.0%), which has earlier been used as stabilizer in phage formulation study (Vinner et al., 2019), and hyaluronic acid (0.2 mg/ml and 1.0 mg/ml), which is commonly used as moisture retention additive in e.g. ophthalmology-related products (Chang et al., 2021). GrowDex diluted with SM buffer (without gelatin) served as control matrix. The compositions were stored in 1 ml volumes at 4°C for up to six months. The stability assay was performed with six model phages: fPoEco01, fTuEco01, fHoEco02, fRuSau02, mEBHT, and KpGranit, representing three phage morphotypes and different genome sizes (**Table 2**).

**Figure 5:**
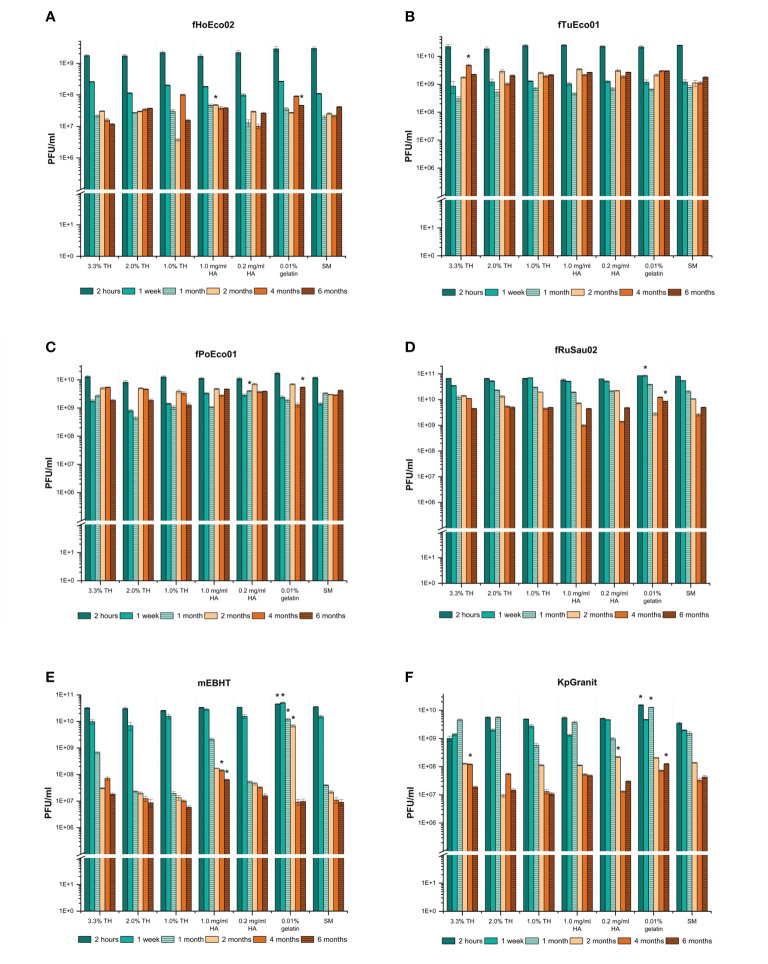
Long-term storage of the phages in tubes. Phages fHoEco02 **(A)**, fTuEco01 **(B)**, fPoEco01 **(C)**, fRuSau02 **(D)**, mEBHT **(E)**, and KpGranit **(F)** were stored in 1.0% (w/v) GrowDex hydrogel containing 3.3%, 2.0%, or 1.0% trehalose (TH), 0.2 mg/ml or 1.0 mg/ml hyaluronic acid (HA) and 0.01% gelatin, or without stabilizer (SM) in 2 ml tubes for six months. Phage titrations were performed in triplicate using the double-layer agar method after 2 hours, 1 week, 1, 2, 4, and 6 months of storage time. Bars and error bars indicate mean and SD of PFU/ml, respectively. Asterix indicates a condition where the phage titer was higher than in other conditions tested at a given time point in statistically significant way, as analyzed by one-way ANOVA (*p*-values ≤ 0.01).

The six model phages behaved very differently in the stability assay. Phage fPoEco01 was the most stable amongst all tested phages, with less than a 10-fold drop in viability across all matrix compositions after six months (**Figure 5C**). However, statistical analysis showed that for fPoEco01, 0.2 mg/ml HA was the best stabilizer at one-month time point and gelatin at six months. Phages fRuSau02 and fTuEco01 were also very stable, showing a 10-fold drop in viability after one month or one week, respectively, and remaining stable after that (**Figure 5B, D**, respectively). Although the differences between stabilizers for these two phages were marginal, the statistical analysis determined that gelatin was one-week and six-month time points for fRuSau02, and 3.3% TH at four months for fTuEco01. Phages fHoEco02 and KpGranit were relatively stable, both suffering ~100-fold drops in viability after one or two months but the titer remained stable after this. For fHoEco02, 1 mg/ml HA was the best stabilizer at two-month time point and gelatin after six months (**Figure 5A**). For KpGranit, statistical analyses suggested a different stabilizers as the best storage medium for different time points. However, the overall stability of KpGranit was the best in 0.01% gelatin, which was the best stabilizer in three time points (**Figure 5F**). Phage mEBHT was the least stable of the tested phages. At time points up to two months, 0.01% gelatin was the best stabilizer and retained the viability of the phage well. With all other stabilizers and without an additional stabilizer, the titer of mEBHT decreased by 100- or 1000-fold in one month (**Figure 5E**). At time points beyond two months, a drastic drop in mEBHT titer from 10^10^ to 10^7^ PFU/ml was observed when gelatin was used as stabilizer. Interestingly, at four-month and six-month time points, 1 mg/ml hyaluronic acid retained the viability of mEBHT better than other stabilizers in statistically significant manner.

**Figure 6:**
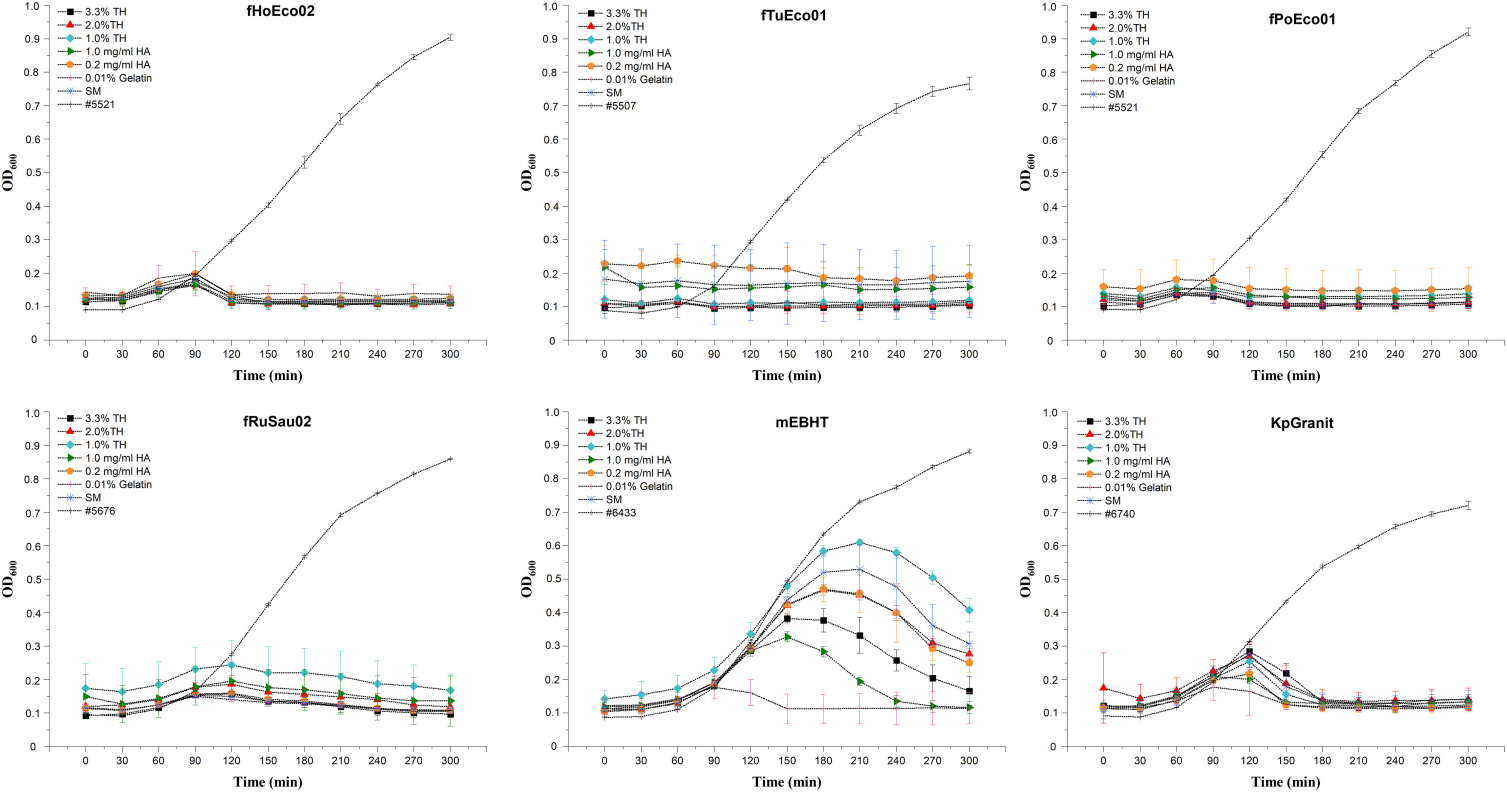
Phage susceptibility test performed with phages stored in phage-hydrogel compositions for six months. Phages fHoEco02, fTuEco01, fPoEco01, fRuSau02, mEBHT, and KpGranit were stored with hydrogel and with either 3.3%, 2.0%, or 1.0% trehalose (TH), 0.2 mg/ml or 1.0 mg/ml hyaluronic acid (HA), 0.01% gelatin, or without stabilizer (SM) in 2 ml tubes for six months. Ten µl of phage mixtures were added into the bottom of microtiter plate wells and 200 µl of diluted overnight bacterial culture was added into the wells. Bacterial growth was followed by measuring optical density at 600 nm in 30-minute intervals for five hours. All measurements were performed in triplicate, and mean and SD values are indicated. Bacterial growth in lysogeny broth without phages served as a control.

Once the stability of phages was analyzed, we aimed to determine whether these phages can be used in phage susceptibility testing after six months of storage. To this end, we used each preparation of stored phage after six months of storage for phage susceptibility testing. Interestingly, phages fPoEco01, fTuEco01, fRuSau02, fHoEco02, and KpGranit still exhibited clear bactericidal activity in all compositions used. As expected, the results with phage mEBHT were more variable. Even though the PST result was considered positive (as judged by 50% inhibition of bacterial growth at the 300-min time point compared against a phage-free control) in all compositions, the clearest positive result was obtained when 0.01% gelatin was used as a stabilizer. Hyaluronic acid (1.0 mg/ml), which was the best stabilizer at six-month time point determined by statistical analysis for mEBHT, was the second-best stabilizer when measured in PST (**Figure 6**).

#### 3.3.2 Shelf-life of the ready-to-screen plates

The phages mixed with hydrogel and chemical stabilizers showed promising results when stored in 1 ml volumes for six months. We investigated whether phages can be directly stored in PST plates for extended periods of time. To study the stability of phages in these ready-to-screen plates, we mixed six model phages fPoEco01, fTuEco01, fHoEco02, fRuSau02, mEBHT, and KpGranit with hydrogel and chemical stabilizers as earlier. The mixtures were added as 10 µl droplets to the bottom of the microtiter plates, which were sealed and stored for six months at 4°C. The plates were tested as PSTs immediately following the preparation, after 1 week, and after 1, 2, 4, and 6 months.

**Figure 7:**
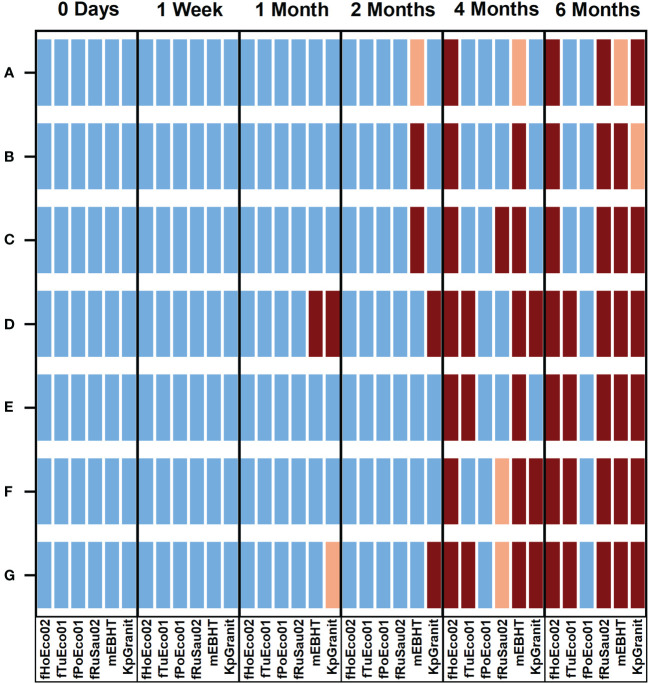
Overview of the stability of phages in ready-to-screen PST plates. Phages fHoEco02, fTuEco01, fPoEco01, fRuSau02, mEBHT, and KpGranit stored with GrowDex hydrogel and with either 3.3% **(A)**, 2.0% **(B)** and 1.0% **(C)** trehalose, 1.0 mg/ml **(D)**, 0.2 mg/ml **(E)** hyaluronic acid, and 0.01% gelatin **(F)**, or without stabilizer **(G)** compositions on ready-to-screen plates as 10 µl droplets for six-months. Ready-to-screen plates were measured using the optimized liquid growth assay protocol developed in this study immediately following plate preparation, as well as after 1 week, 1, 2, 4, and 6 months of storage time. Bacterial growth was followed by measuring optical density at 600 nm for five hours. All the measurements were performed in triplicate, and mean and SD values were calculated. Bacterial growth in lysogeny broth without phages served as a control. Result interpretations are color-coded. Blue: Phage was viable. PST gave clear positive results and bacterial growth was < 50% of the control. Orange: Phage was partially viable;. bacterial growth was 50 to 70% of the control. Brown: Phage was not viable; bacterial growth was > 70% of the control.

We found that phage fPoEco01 was the most stable when stored as 10 µl droplets. The shelf-life of this phage was at least 6 months in all the tested mixture compositions, as shown in **Figure 7**. The second-best shelf-life was obtained for phage fTuEco01, which was infective towards its host for four months when stored with 0.01% gelatin and 6 months when stored with either 3.3%, 2.0%, or 1.0% trehalose. Phage fHoEco02 remained infectious for two months, after which the phage lost its infectivity in all tested compositions. Phage fRuSau02 was infective with 3.3% and 2.0% trehalose, and 1.0 mg/ml and 0.2 mg/ml hyaluronic acid for four months. KpGranit stayed stable with all trehalose concentrations and 0.2 mg/ml hyaluronic acid for up to four months and in 0.01% gelatin for two months. However, in 1.0 mg/ml hyaluronic acid and SM-buffer, KpGranit lost its viability already in one month. Phage mEBHT remained infectious for two months in SM, 0.01% gelatin, and both concentrations of hyaluronic acid, but only for one month if trehalose was included in the mixture.

To conclude, we observed that all phages were stable and remained viable for two months, when either 0.01% gelatin or 0.2 mg/ml hyaluronic acid was added to the composition. None of the tested stabilizer compounds were universally suitable for all phages when stored for longer than two months. Nevertheless, both 0.01% gelatin and 0.2 mg/ml hyaluronic acid were suitable chemical stabilizers that can give ready-to-screen plates a shelf-life of two months.

#### 3.3.3 Transportation of the ready-to-screen plates

**Figure 8:**
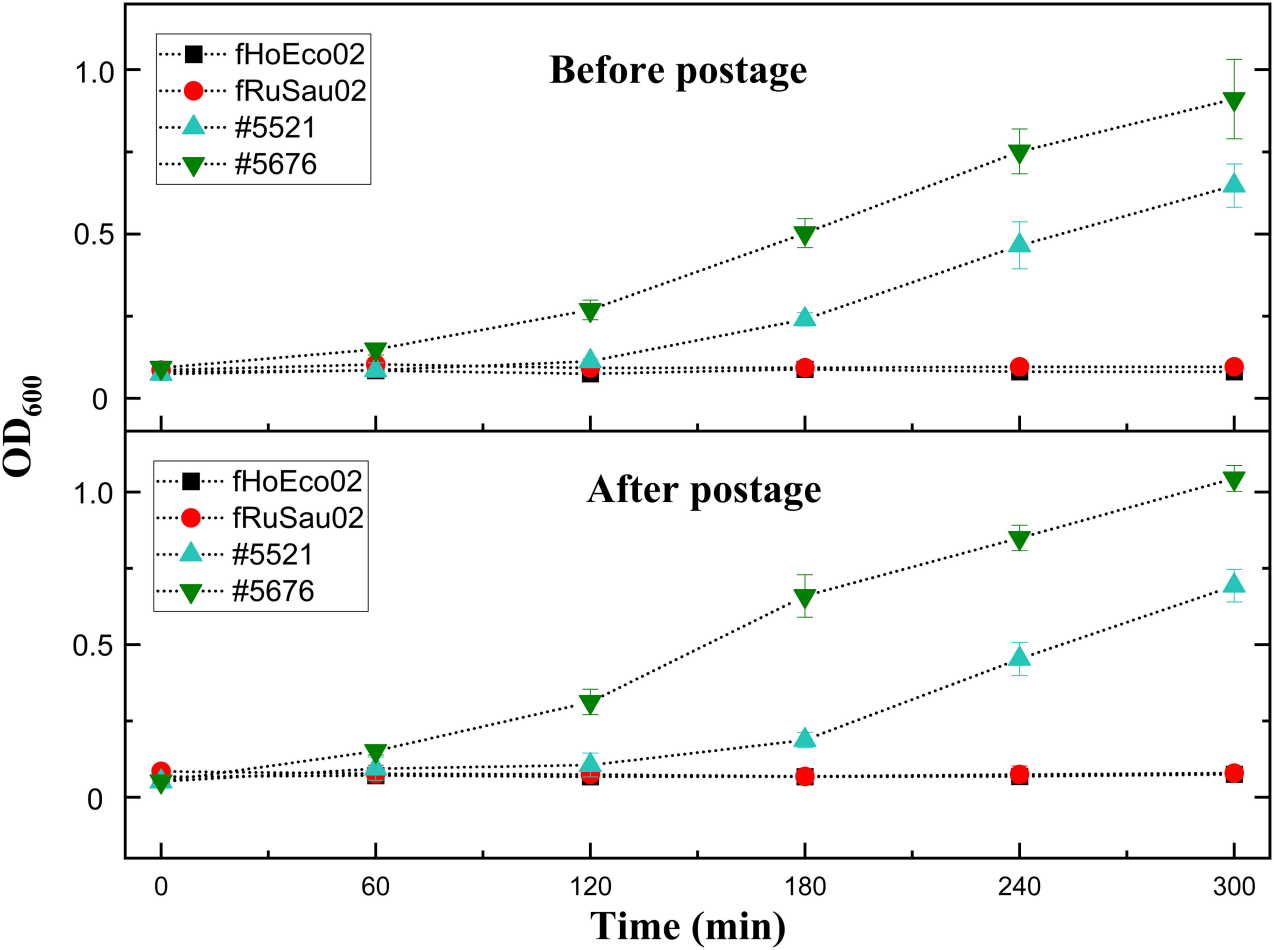
Phage transportation in ready-to-screen plates. Phages fHoEco02 and fRuSau02 were mixed with hydrogel and 10 droplets were added to the bottom of the microtiter well plates. Plates were then sealed and one of the plates was tested immediately after preparation. The other one was sent *via* post back to ourselves and tested after one week after its preparation. Phage viability was tested with the corresponding host strains #5521 (fHoEco02) and #5676 (fRuSau02). The measurement was done utilizing optimized liquid assay and absorbance reader.

As phage collections of individual phage therapy/research laboratories are not always broad enough, collaboration between laboratories is essential. Thus, the transportation of phages between laboratories is one of the key elements to the successful selection of phages for phage therapy. While ready-to-screen plates were found to possess a shelf-life of two months in our laboratory, we wanted to test whether hydrogel-based ready-to-screen plates can be transported safely *via* mail. To this end, we first tested the transportation to ourselves to see if there would be any stability issues due to harsh conditions during the shipment. We prepared two identical plates using phages fHoEco02, fTuEco01, fRuSau02, and mEBHT embedded with hydrogel, and tested their infectivity with their hosts #5521, #5507, #5676, and #6433, respectively. One of the plates was tested immediately following preparation, and another plate was tested one week later, after it was mailed back to ourselves. The two plates gave identical results (**Figure 8**), indicating that phages survived the transportation in ready-to-screen plates. We then verified this result by sending similar ready-to-screen plates to the phage research laboratory in DSMZ, Germany. Also in this test, phages were viable and gave positive results in the PST assay after the transportation, approx. one week after the preparation of the plates (not shown).

## 4 Discussion

Phage therapy has been practiced for more than 100 years, but it declined in popularity following the commercialization of antibiotics. However, the current global crisis surrounding the increase in antibiotic resistance has revived global interest in phage therapy (Clokie MR et al., 2011). As the need for phage therapy may increase in coming years, there are many challenges that must be overcome to provide safe, effective, and expedient phage therapy for patients (Opperman et al., 2022).

When performing personalized phage therapy, rapid PST screening of hundreds or even thousands of phages may be required in order to identify phages that are well suited to treat the infection of a particular patient (Schooley et al., 2017; Dedrick et al., 2022). With the help of fast and high throughput phage screening methods, routine phage diagnostics can be done efficiently (Henry et al., 2012; Rajnovic et al., 2019).

In this study, we developed a liquid culture PST method utilizing hydrogel as a phage matrix. The method is suitable for traditional OD readers as well as for a microscopy reader. With an OD reader, the assay needed an overnight culture of host bacteria, after which the test itself could be run within five hours. If microscopy reader was used, most bacterial species tested did not need any pre-culture step, and the assay could be performed in just three hours. However, it should be noted that five- or three-hour assay times are unlikely to capture the emergence of possible phage-resistant bacterial mutants present in a particular bacterial strain population. Therefore, if the rise of phage resistance is to be observed, the assay time must be increased.

The optimal bacterial starting concentration in the PST assay varied clearly between different bacterial species but was fairly consistent among different strains of each tested species. In the future, it would be interesting to test this method with very slow-growing bacterial species such as *Mycobacterium* spp. The high sensitivity of microscopy readers may accelerate PSTs of these fastidious organisms. In this work, the input MOI values of phages in the PST were below 1. The use of low MOI values is essential in order to verify that a positive result is not due to lysis from without but reflects a true phage infection.

For the PST to be robust and fast, the plates containing phages should ideally be prepared beforehand. We hypothesized that hydrogels might be used as phage matrices to prevent phages from drying and thereby maintain the phages’ viability in ready-to-use microtiter plates. To test this hypothesis, we evaluated the potential of four commercial hydrogels (Askina, Purilon, GrowDex, and Intrasite) to be used as phage matrix. Askina and Purilon were found to inhibit bacterial growth, which would lead to false positive results in PSTs, and were thus excluded from further study. GrowDex and Intrasite hydrogels did not inhibit bacterial growth and were selected for short-term phage storage experiments. Both hydrogels maintained phage viability, which is a similar result to earlier work by Merabishvili et al., who found that the viability of five phages was retained in Intrasite for a 24-hour assay time (Merabishvili et al., 2017). However, in our work, the phage stability in GrowDex was clearly greater than in Intrasite. The two hydrogels possess slightly different chemical compositions, as GrowDex is composed of nanofibrillar cellulose and Intrasite of carboxymethyl cellulose. Nanofibrillar cellulose in GrowDex is in native form, neutral in charge and inert material, whereas carboxymethyl cellulose is produced by chemical modifications. These properties may explain their different behavior with phages. GrowDex has also been demonstrated to be a highly biocompatible culturing matrix for various cell types and it is widely used in three-dimensional cell culture, tissue explant culture, and drug release studies (Lauren et al., 2014; Lou et al., 2014; Chang et al., 2020). To our knowledge, this is the first study using GrowDex as matrix for bacteria and bacteriophages.

Earlier studies have analyzed the stability of phages in hydrogels and polymers from therapeutic delivery aspect (Malik et al., 2017). Compounds like Intrasite, Fibrin Glue, and Defensive Antibacterial Coating (DAC) hydrogel have been used with phages with variable results (Merabishvili et al., 2017; Rubalskii et al., 2019; Ferry et al., 2020). However, the focus in these studies was the short-term stability of phages and the possibility to use the compounds as delivery vehicles in therapeutic applications, and the antibacterial nature of the compounds was not considered a restriction. To our knowledge, this is the first time when hydrogels have been tested as phage matrices in PST.

We also attempted to further improve phage stability in GrowDex by supplementing the phage matrix with additional stabilizers (3.3%, 2.0%, and 1.0% trehalose, 1.0 and 0.2 mg/ml hyaluronic acid, and 0.01% gelatin) in the compositions, we noticed that different phages behaved very differently in the applied conditions. When storing the phage-hydrogel mixtures in 1 ml volumes in tubes, all other phages except mEBHT were still viable and returned positive results in a PST after six months of storage in all hydrogel compositions. However, the least stable phage, mEBHT belonging to the genus *Rosenblumvirus*, displayed clear infectivity only when supplemented with 0.01% gelatin or 1.0 mg/ml hyaluronic acid as stabilizers. In other stabilizers and without stabilizer, the phage viability was lower, and the result of the PST was less clear. The variation in phage stability with different stabilizers was more apparent when phage-hydrogel mixtures were stored in the bottom of microtiter plate wells as “ready-to-screen” plates. In these conditions, all tested phages survived for two months in mixtures containing 0.01% gelatin or 0.2 mg/ml hyaluronic acid. However, the viability of phages mEBHT and KpGranit began to decrease already after 1 month in mixtures containing 0.2 mg/ml hyaluronic acid and without an additional stabilizer. We were not able to increase the stability of all tested phages beyond two months in any tested condition. We hypothesize that the difference in phage stability in tube and plate storage is due to drying of the 10 µl droplets when stored for longer than two months. Perhaps surprisingly, we found that trehalose is not an effective stabilizer in our study, despite earlier studies finding it to be an effective stabilizer to maintain phage viability during freeze-drying (Merabishvili et al., 2013; Ly et al., 2019; Malik, 2021). This discrepancy may be explained by the fact that in our study, different phages reacted to trehalose in very different ways. For example, phages fPoEco01, KpGranit, and fTuEco01 remained fully infectious in trehalose in ready-to-screen plates for four to six months, whereas mEBHT was inactivated under the same conditions within just two months.

The phages used as models in the stability assays represent different morphologies and sizes. Two of the phages, fHoEco02 and fRuSau02, have myovirus morphologies and large genomes sizes (167.0 kb and 148.5 kb, respectively). Although these two phages remained infectious for six months when stored in tubes, they were relatively unstable when stored in small volumes in ready-to-screen plates. Out of the two phages having siphovirus morphology, KpGranit has a 122.7 kb genome and fPoEco01 a 44.4 kb genome. Interestingly, fPoEco01 was found to be the most stable phage in the study; it remained fully functional in all tested conditions for six months even in ready-to-screen plates. KpGranit, on the other hand, was relatively unstable and showed a clear response to the stabilizer used; the phage began to lose its infectivity in ready-to-screen plate within one month in GrowDex containing 0.2 mg/ml hyaluronic acid and without an additional stabilizer but remained viable for four months when trehalose or gelatin were used as stabilizers. The two phages with podovirus morphology, fTuEco01 (44.3 kb genome) and mEBHT (17.5 kb genome), also behaved differently. fTuEco01 was a fairly stable phage and survived in ready-to-screen plates for six months when trehalose was used as stabilizer. mEBHT, on the other hand, was the least stable phage in our study and survived for only two months in ready-to-screen plates. Further, this phage appeared to be inhibited by all three tested concentrations of trehalose, as phage viability decreased after two months in mixtures containing trehalose relative to all other conditions, including GrowDex without stabilizer. Although the number of phages assessed for stability in ready-to-screen plates was very small (six phages), it is apparent that phage morphology or genome size cannot be used as reliable measures to predict phage stability or response to different stabilizing agents.

One of the interesting findings in this work was that ready-to-screen plates containing GrowDex and gelatin as phage matrix can be used for phage transportation between laboratories. We also demonstrated that phages remained infectious for at least one week when stored at RT in GrowDex. These findings open new possibilities for collaboration between different laboratories, as transportation of phages in ready-to-screen plates reduces the time required to start the PST in the receiving laboratory.

## 5 Conclusions

In this work, we developed a rapid, shelf-stable PST which utilizes nanofibrillar cellulose hydrogel and gelatin as a phage storage matrix. The assay time when measuring bacterial growth with a microscopy reader was as short as three hours, and there was no need for preparing overnight pre-cultures of clinical bacteria prior the assay. All phages tested remained viable in hydrogel mixture for at least six months when stored in 1 ml volume and for two months in “ready-to-screen” plates as 10 µl droplets. The use of hydrogel as a phage storage matrix in ready-to-screen plates will enable rapid high-throughput screening of phages for personalized phage therapy.

## Data availability statement

The original contributions presented in the study are included in the article/**Supplementary Material**. Further inquiries can be directed to the corresponding author.

## Author contributions

SP, LP, MS, and SK conceptualized the study. SP and SK designed the work. SK supervised the study. SP, ES, and AD performed the experiments. SP, ES, AD and SK analyzed the data. SP and SK wrote the manuscript. All authors revised and accepted the manuscript. All authors contributed to the article and approved the submitted version.
