# Supplementary material for: Rapid hydrogel-based phage susceptibility test for pathogenic bacteria
Source: Front Cell Infect Microbiol. 2022 Dec 7;12:1032052. doi: 10.3389/fcimb.2022.1032052 (PMC9771388; doi:10.3389/fcimb.2022.1032052)
Supplement: Supplementary file 1 [file DataSheet_1.docx]

**SUPPLEMENTARY MATERIAL**

**Figure S1. Phage optimization for liquid growth assay.**

**Figure S2. Growth curves of eight bacterial species and their respective phages mixed with hydrogel.**

**Table S1: CFU and MOI values used in the work.**

**
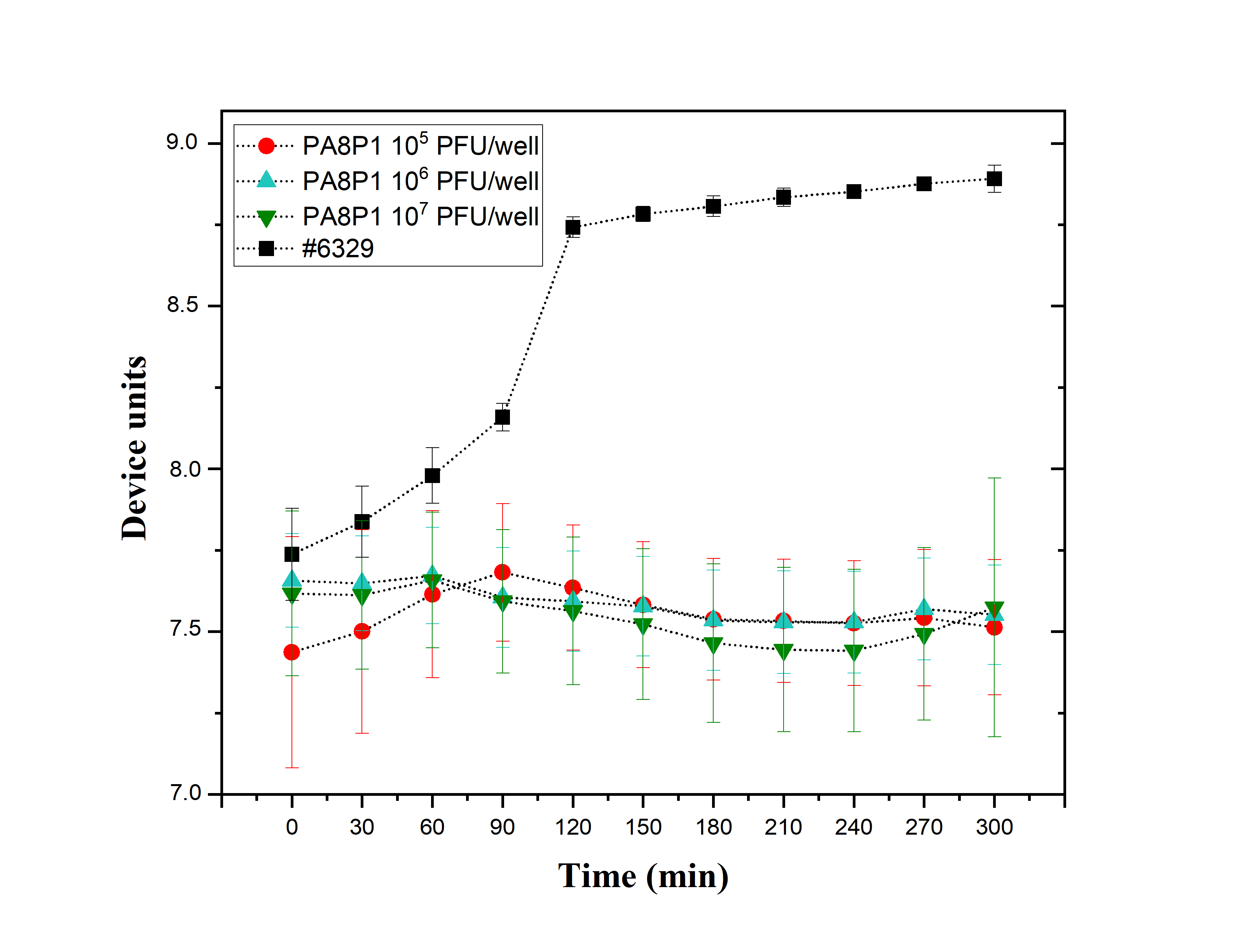
**

**Figure S1. Phage optimization for liquid growth assay.** Growth curve of *P. aeruginosa* strain #6329 infected with phage PA8P1 in three different PFU/well amounts (10^5^, 10^6^, and 10^7^). Bacteria without phage (#6329) served as control. Phage was aliquoted as 10 µl droplets into the bottom of microtiter well plate. Bacteria were cultured overnight, adjusted to OD 0.35, diluted 1:100, and 200 µl of the dilution was added on top of the phage. Phage growth was measured using liquid culture assay with microscopy reader. Bacterial growth was followed in 30-minute intervals for five hours. Device units indicate the microscopy reader values. Measurements were done in triplicates, mean values and SD are indicated.

**
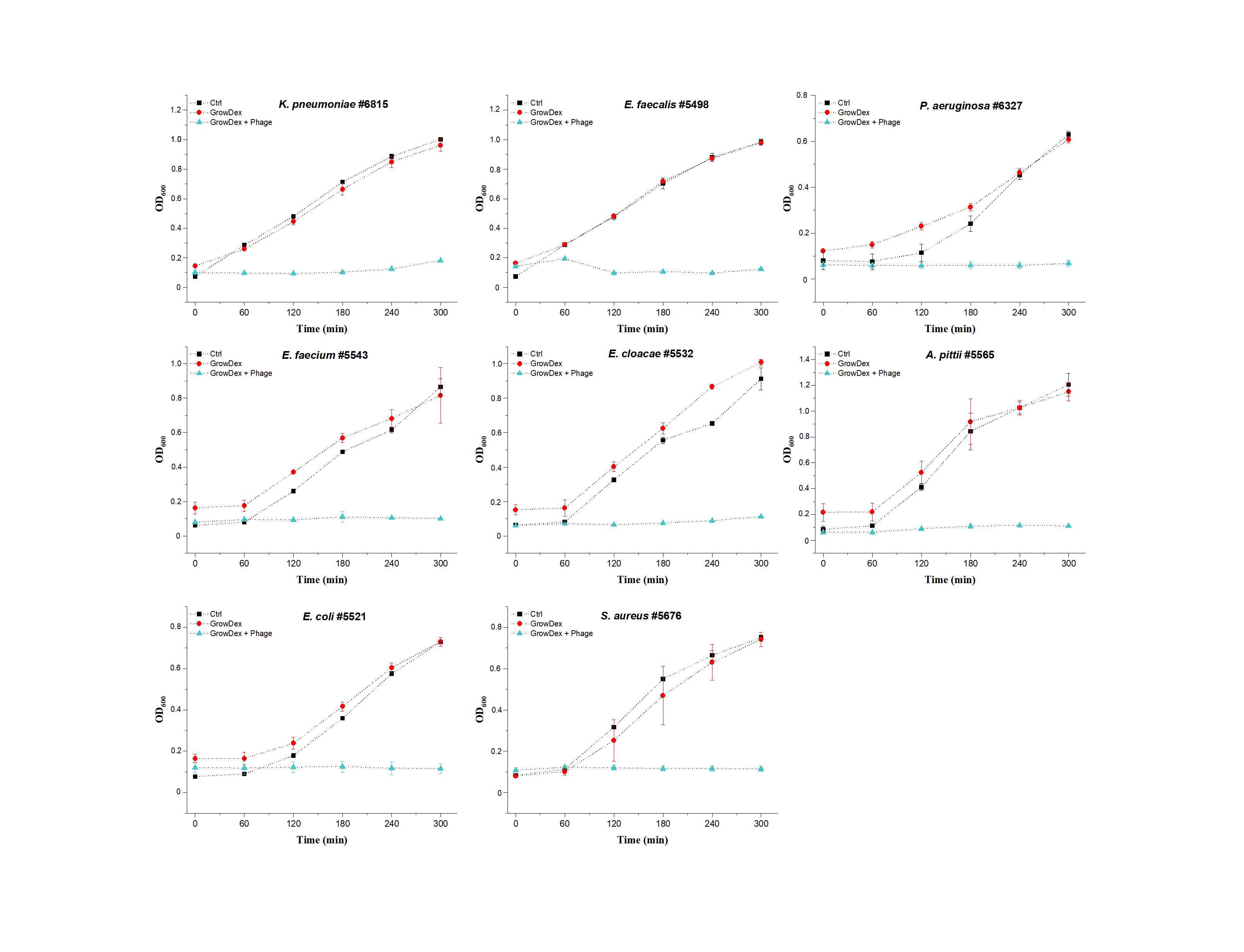
**

**Figure S2. Growth curves of eight- bacterial species and their respective phages mixed with hydrogel.** Growth curves of bacterial strains *K. pneumoniae* #6815, *E. faecalis* #5498, *P. aeruginosa* #6327, *E. faecium* #5543, *E. cloacae* #5532, *A. pittii* #5565, *E. coli* #5521, and *S. aureus* #5676 tested against their infective phages vB_KoM-Pickle, F-Mali04, PA1P1, fHoEfm06, fOuEcl03, fHyAci03, fHoEco02, and fRuSau02, respectively. Phages were mixed with GrowDex hydrogel and the mixture was added into bottom of the microtiter well plate as 10 µl droplets. Overnight bacterial culture was diluted and 200 µl was added into the wells. Bacterial growth was followed by measuring optical density at 600 nm in one-hour intervals for five hours. All of the measurements were performed in triplicates and mean and SD values are indicated. Bacterial growth in lysogeny broth served as control.

**Table S1.** CFU and MOI values used in the work. MOI values refer to phages infecting the corresponding strains as described in Table 2.

| Species | Strain code | Overnight culture  CFU/ml | OD reader  starting CFU | OD reader  MOI input | OD 0.35  CFU/ml | Microscope starting CFU | Microscope  MOI input |
| --- | --- | --- | --- | --- | --- | --- | --- |
| *A. pittii* | #5565 | 4.5 × 10^9^ | 9.4 × 10^6^ | 0.1 | 1.1 × 10^8^ | 1.1 × 10^5^ | 0.9 |
| *E. cloacae* | #5532 | 5.9 × 10^9^ | 1.2 × 10^7^ | 0.08 | 5.8 × 10^8^ | 5.8 × 10^5^ | 0.2 |
| *E. faecalis* | #6569 | 8.2 × 10^9^ | 3.3 × 10^7^ | 0.3 | 5.2 × 10^8^ | 1.0 × 10^6^ | 0.01 |
| *E. coli* | #5521 | 3.7 × 10^9^ | 1.5 × 10^6^ | 0.7 | 4.3 × 10^8^ | 4.3 × 10^5^ | 0.002 |
| *K. pneumoniae* | #6740 | 9.2 × 10^9^ | 3.7 × 10^6^ | 0.3 | 3.8 × 10^8^ | 3.8 × 10^5^ | 0.3 |
| *P. aeruginosa* | #6728 | 4.9 × 10^9^ | 2.5 × 10^7^ | 0.4 | 5.7 × 10^8^ | 1.1 × 10^6^ | 0.09 |
| *S. aureus* | #5676 | 4.4 × 10^9^ | 8.8 × 10^6^ | 0.1 | 4.1 × 10^8^ | 4.1 × 10^5^ | 0.2 |
